# Safety and efficacy of electroconvulsive therapy during pregnancy: A systematic review

**DOI:** 10.1192/j.eurpsy.2025.2125

**Published:** 2025-08-26

**Authors:** A. Karmous, H. Ghabi, N. Karmous, R. Lansari, A. Larnaout, W. Melki

**Affiliations:** 1 Department D; 2 Department G, Razi hospital, Manouba; 3 Gynaecology and obstetrics department B, Charles Nicoles hospital, Tunis, Tunisia

## Abstract

**Introduction:**

Pregnancy and the immediate postpartum period are at high risk of decompensating or developing psychiatric disorders. Electroconvulsive therapy (ECT) is an effective treatment for severe and resistant mental disorders and could be a therapeutic option for psychiatric disorders during pregnancy.

**Objectives:**

The aim of our study was to investigate the safety and the efficacy of ECT in pregnant women through a systematic review.

**Methods:**

A systematic review was conducted. PubMed via Medline, Google Scholar and Semantic Scholar were used as search engines. The keywords used were (“Electroconvuslive therapy” or “ECT”) and (“pregnancy” or “pregnant”). Clinical trials, case reports and case series assessing the efficacy and safety of ECT during pregnancy, published from inception to December 2023 and written in English or Frensh were included.

**Results:**

A total of 30 articles were included for the final analysis with a total of 96 cases. The mean age of patients was 30.1 years. ECT was mostly performed during the first trimester of pregnancy with 45.3% of patients. The main psychiatric diagnoses were major depressive disorder with 47.6% of patients, followed by bipolar disorder with 19.3%. The average number of sessions performed was 10.4 with a maximum of 22. A partial improvement or a total resolution of symptoms were noted in 78.6% of cases. Transient fetal arrhythmia (not requiring drug intervention) was the most common complication, occurring in 6.25% of cases (n=6). Fetal death or abortion were observed in 4.1% of cases (n=4).

**Conclusions:**

ECT appears to be an effective treatment for severe psychiatric disorders in pregnant women. However, it needs to be performed as part of a multidisciplinary care team to reduce the risk of serious consequences for both the mother and the fetus.

**Disclosure of Interest:**

None Declared

